# Solvent System-Guided Extraction of *Centaurium spicatum* (L.) Fritch Provides Optimized Conditions for the Biological and Chemical Characteristics of the Herbal Extracts

**DOI:** 10.3390/ph16020245

**Published:** 2023-02-06

**Authors:** Jelena Božunović, Marija Ivanov, Jovana Petrović, Uroš Gašić, Đura Nakarada, Milica Milutinović, Neda Aničić, Zlatko Giba, Danijela Mišić, Dejan Stojković

**Affiliations:** 1 Institute for Biological Research “Siniša Stanković”—National Institute of the Republic of Serbia, University of Belgrade, Bulevar despota Stefana 142, 11060 Belgrade, Serbia; 2 Faculty of Physical Chemistry, University of Belgrade, 11158 Belgrade, Serbia; 3 Faculty of Biology, University of Belgrade, 11000 Belgrade, Serbia

**Keywords:** *Centaurium spicatum*, extraction solvent system, bioactivity, LC/MS

## Abstract

Spiked centaury (*Centaurium spicatum*) is a well-known medicinal plant from the Mediterranean region with various bioactivities, but there are no studies addressing the use of different solvent systems to improve its pharmacological potential. Nine extraction procedures were adapted to study the effects of solvent composition on the content of bioactive compounds in *C. spicatum* extracts and on corresponding bioactivities. Targeted metabolomics was performed to obtain information on the chemical composition of extracts. Ethanol-water-based extraction procedures were the most efficient in isolating polyphenols, while less polar butanol extract contained the highest amount of iridoids. Antioxidant potential analysis revealed stronger activity in extracts with higher polyphenol content. *Bacillus cereus* and *Staphylococus aureus* were designated as the most sensitive bacterial strains to the activity of extracts, while among the micromycetes tested, *Penicillium funiculosum* was the most susceptible strain. Butanol extract showed antivirulence potential on *Candida albicans* morphological transition from yeast to hyphal form, and selected extracts were effective against biofilm formation in two *Candida* species. All the extracts tested in this study showed no cytotoxic activity to immortalize human skin keratinocyte cell line (HaCaT), whereas extracts obtained by ethanol-water extraction stand out for their potent wound healing effects. Moreover, the influence of the extraction solvent system on various bioactivities of *C. spicatum* is reported herein for the first time. Overall, the results presented in this study promote the use of *C. spicatum* as a source of natural products with potential antioxidant, wound healing, and antimicrobial applications that are potentially safe for human use.

## 1. Introduction

In recent decades, antimicrobial resistance has become a global health-related concern worldwide, considering the increasing resistance of bacteria and fungi to commercial antibiotics and antifungals. With the continuous increase in microbial resistance in agriculture and medicine, the development of new antimicrobial agents has become an essential research task. Bacterial resistance to commercial antibiotics is also one of the reasons for the emergence of resistant bacterial biofilm communities that contribute to chronic infections. These structured communities (biofilms) of bacteria and fungi communicate via a quorum-sensing system and are embedded in an extracellular matrix that enables them to overcome the action of antimicrobial agents. Therefore, the detection of new antimicrobial agents that could end this emerging battleground is an overriding research challenge [1]. Furthermore, in vitro cytotoxicity screening of new agents with antimicrobial potential must be an accompanying step in this research since it determines the potential safety of newly developed products and allows their possible medical use. Keeping in mind that oxidative stress (overproduction of free radicals without adequate neutralization) is held responsible for several chronic illnesses, including cancer, diabetes, and cardiovascular disease, the survey for compounds with antioxidant properties has become a trend of its own.

In this regard, plants are noteworthy, representing a rich source of diverse antioxidant and antimicrobial compounds, also known as specialized metabolites, such as tannins, flavonoids, alkaloids, and terpenoids [2,3,4] that are not available in synthetic compound libraries [5].

There are an estimated 250,000 plant species in the world, and only 5–15% have been tested as potentially pharmacologically useful [6]. There is encouraging potential for the discovery of plant metabolites with useful pharmacological activities for the treatment of a variety of diseases in humans and animals.

**Figure 1:**
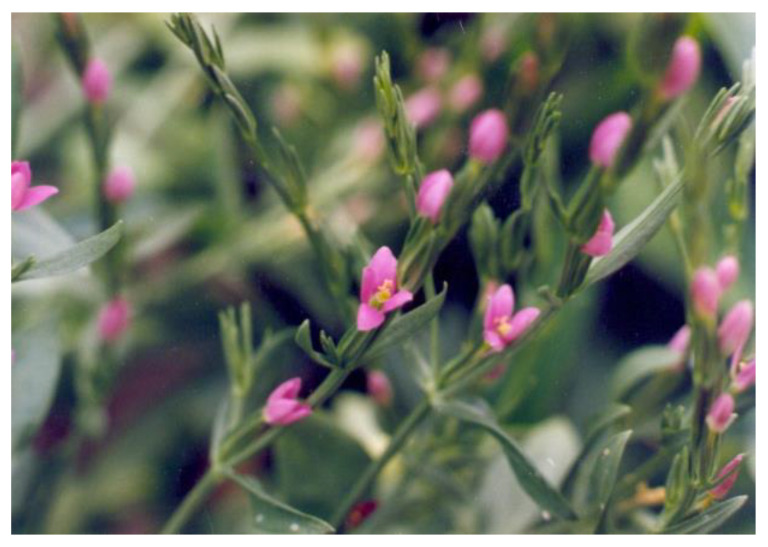
*Centaurium spicatum* flowering tops of individuals from nature.

*Centaurium spicatum* (L.) Fritch (syn. *Schenkia spicata* L. Mansion) is a common Gentianaceae species of the Mediterranean region and Eastern Europe, where it is adapted to various saline soils and is capable of completing life cycles under very harsh environmental conditions [7]. Spiked centaury (Figure 1.) is a rich source of pharmacologically active specialized metabolites, including secoiridoid glucosides (sweroside, swertiamarin, and gentiopicrin) and phenolic compounds. Because of the bitterness of secoiridoid glucosides, these compounds are used in the preparation of some commercial beverages [7]. The extract of *C. spicatum*, along with other *Centaurium* species such as *C. pulchellum*, has been used in traditional medicine for the treatment of abdominal pain, hypertension, kidney and ureteral stones, renal colic, wounds, and diabetes [8]. The compounds found in the methanol extract of *C. spicatum* showed cytotoxic activities against three different types of human cancer cell lines, HeLa, THP-1, and HL-60 [9]. Recent studies have shown that lisianthoside II, together with seven other secoiridoid glycosides from spiked centaury, may be a suitable basis for the development of new drugs against COVID-19 [10].

It has been previously reported that the optimization of extraction conditions can affect the biological potential of extracts from plant species belonging to the Gentianaceae family [11,12,13,14]. Besides the extraction methods and conditions, such as type of solvent, solvent composition, temperature, extraction, time, and number of extraction steps [15,16], the composition of bioactive compounds in plant extracts depends on several other factors, such as plant physiological stage and geographical origin [17].

The present study was conducted to determine the most efficient extraction solvent system on the comprehensive recovery of specialized metabolites from *C. spicatum* herbs, with the aim to optimize the pharmacological effects of extracts. In order to achieve this, a targeted metabolomics method was used to characterize nine extracts of spiked centaury using UHPLC/DAD/QqQMS/MS. The next objective was to screen these extracts for their bioactive properties, i.e., to evaluate their: (1) antimicrobial, (2) antibiofilm, (3) antioxidant, and (4) cytotoxic properties in a keratinocyte cell line (HaCaT). The data reported here could be of great importance for the facilitated use of *C. spicatum* extracts as food preservatives and nutraceuticals.

## 2. Results and Discussion

The aim of this study was to investigate the influence of different extraction procedures on the chemical composition of *C. spicatum* extracts and on the resulting biological activities.

### 2.1. Extraction Yields

The yields of the nine extracts analyzed are shown in Table 1. The lowest value was obtained for sample Cs6 (10.0% for extraction with 100% butanol), whereas the highest was obtained for extract Cs9 (29.5%, for the buthanol:ethanol 30:70 extract), followed by the extract Cs3 (25.0%, extraction with ethanol:water 50:50).

### 2.2. Different Solvent Systems Affect the Chemical Profile of C. spicatum Extracts

Chemical profiles of spiked centaury extract determined by the UHPLC/(±)HESI-QqQ-MS/MS targeted metabolomics analysis revealed data on phenolic acids, flavonoid glycosides, and flavonoid aglycones, as presented in Table 2.

Regarding phenolic compounds, among the tested extracts, Cs2 had the highest share of the identified polyphenolic compounds (187.10 mg/kg), followed by Cs3 and Cs4 extracts, all of which were ethanol:water extracted. In general, rutin was the most dominant compound in the extracts (3.32–125.25 mg/kg) and was followed by caffeic acid (5.95–20.68 mg/kg) and eriodictyol (6.98–9.69 mg/kg). Rutin was the dominant compound in all extracts analyzed except for the 100% water extract Cs5, which was characterized by the highest proportion of caffeic acid. The obtained results are in accordance with previously published data on the phytochemical profile of common centaury (*Centaurium erythraea*), which also revealed the presence of secoiridoid glucosides and phenolic compounds as the main constituents [18,19,20]. Flavonoid glycosides were previously detected in extracts of *C. spicatum* [21,22]. Our current study showed that these compounds, along with phenolic acids, were effectively extracted using the combination of polar solvents, such as ethanol:water, while flavonoid aglycones luteolin, quercetin, naringenin, and isorhamnetin were the most abundant in butanol extracts. In agreement with our results, previous chemical studies of the methanol extract of *C. erythraea*, *C. littorale*, and *C. pannonicum* revealed that rutin was the most abundant flavonoid compound, with mean amounts ranging from 1.7 µg/g dry weight (dw) in *C. erythraea* to 30 µg/g dw in *C. littorale* [23].

Among the iridoid compounds, secoiridoid sweroside was the most abundant in spiked centaury extracts, with the highest concentration in butanol extract Cs6 (62.89 mg/kg). Extracts Cs7, Cs8, and Cs9 contained the highest amount of gentiopicrin, while 100% ethanol (Cs1) was the most efficient solvent for the extraction of swertiamarin. The secoiridoid glycosides sweroside, swertiamarin, and gentiopicrin were previously described as the major specialized metabolites of the genus *Centaurium* [19,23,24,25].

Overall, it may be concluded that the chemical profile of *C. spicatum* extracts is highly determined by the type of solvent used for the initial extraction.

### 2.3. Antioxidant Activity

#### 2.3.1. ABTS and FRAP Free Radical Scavenging Activity Assay

Based on the results obtained by radical scavenging capacities in ABTS and FRAP assays, we found the strongest antioxidant potential in ethanol:water extracts Cs2, Cs3, and Cs4 (Table 3). The designated extracts contained also the highest amount of polyphenolic compounds, in the range of 142.78–187.10 mg/kg. Since two of the most abundant molecules, rutin and caffeic acid, are potent antioxidants [26,27,28], the prominent antioxidant potential of extracts Cs2, Cs3, and Cs4 may be attributed to their high content of phenolic compounds as well. Nevertheless, the lowest antioxidant activity was observed in water extract Cs5, which was the poorest polyphenol-containing extract (50.05 mg/kg).

#### 2.3.2. Determination of Scavenging Activity toward DPPH Radicals

Electron paramagnetic resonance (EPR) spectroscopy was used to study the antioxidant activity of selected water-insoluble compounds toward biologically relevant free radicals.

**Figure 2:**
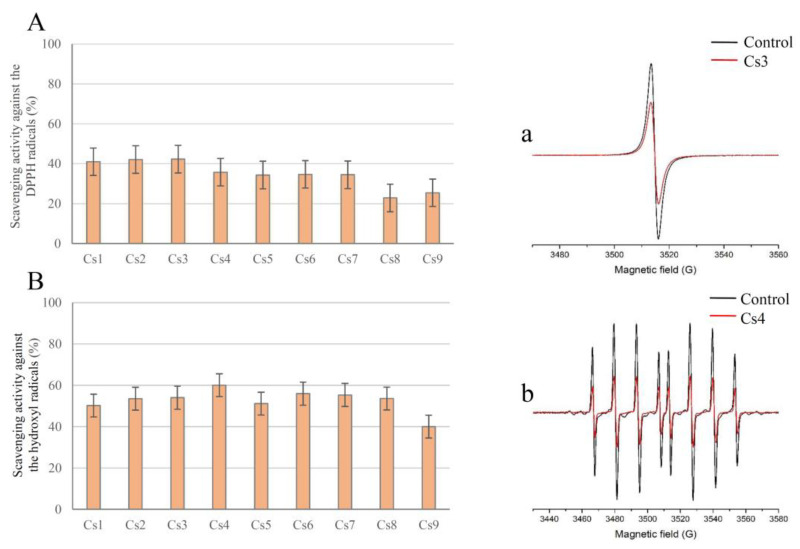
Anti-DPPH radical activity (%) 2 min upon the addition of *C. spicatum* extracts into the system (**A**) and the anti-^•^OH radical activity (%) 2 min upon the ^•^OH generation in the system containing *C. spicatum* extracts (**B**). Representative EPR spectra of (**a**) DPPH radical and (**b**) DEPMPO/OH spin adducts in Cs3 (**a**) and (**b**) Cs4 extracts.

The EPR spectra obtained 2 min after the interaction of DPPH with the extracts of *C. spicatum* are shown in Figure 2.

Results obtained in this study indicate that the ethanol-water extracts have the highest DPPH scavenging potential, which is in agreement with the results obtained in ABTS and FRAP tests and suggests the overall significant radical scavenging activity of the *Centaurium* extracts.

#### 2.3.3. Determination of the Scavenging Activity toward the Hydroxyl Radicals

To determine the amount of OH radicals, the DEMPO spin trap was chosen due to its suitable selectivity and long spin-adduct half-life (132 min for DEMPO/OH [29]). The results of the EPR spectra of OH/DEPMPO spin adducts obtained 2 min after the generation of ^•^OH radicals in the system containing *C. spicatum* extracts are presented in Figure 2. The anti-^•^OH activities (AB) of the *C. spicatum* extracts were calculated using the previously described formula. The calculated anti-OH activities of the extracts are shown in Figure 2.

Based on the results of this experiment, it can be concluded that all samples showed similar scavenging activity against ^•^OH radicals, with Cs4 extract having the strongest ^•^OH radical scavenging activity. Contrary to Cs4, Cs9 extract was found to have the lowest potential to eliminate ^•^OH radicals.

### 2.4. Antibacterial Activity against Gram-Positive and Gram-Negative Bacteria

All nine extracts of *C. spicatum* showed suitable activity toward three tested Gram-positive and three Gram-negative bacterial strains (Table 4). These bacterial strains belong to the group of foodborne pathogens that can cause infections in humans and animals. The overuse of commercial antibiotics is accelerating antibiotic resistance in various human pathogens and points out the need to identify new natural products, such as molecules of plant origin with antibacterial potential.

The antibacterial capacity of extracts Cs7, Cs8, and Cs9 against Gram-negative bacteria, *Salmonela* Typhimurium, *Escherichia coli*, and *Enterobacter cloacae*, may be related to the presence of flavonoid compounds, namely quercetin, naringenin, and isorhamnetin, whose antibacterial potential has been described previously on several occasions [30,31,32]. In the current study, extract Cs1, the extract with the highest swertiamarin content, was the most effective in reducing the growth of *Listeria monocytogenes*, displaying an MIC of 0.5 and MBC of 1 mg/mL, while extract Cs2 was more effective in reducing the growth of Gram (+) bacteria than of Gram (−) bacteria. Our study is in agreement with a previous report by Bozunovic et al. [18] showing that common centaury methanol extract with high content of swertiamarin was also effective against *Listeria monocytogenes*. *Bacillus cereus* and *Staphylococus aureus* were the most susceptible strains to the activity of the tested extracts (MIC values 0.5 mg/mL for extracts Cs4 and Cs5), whereas *S.* Typhimurium and *E. cloacae* were the most resistant bacterial strains (MIC ranges of 1–2 mg/mL). Previously published data [19] on the antibacterial potential of methanol extracts from the aerial parts of *C. spicatum* showed lower MIC values than in this study (0.1–0.25 mg/mL). These differences could be attributed to different solvents used for the extraction and the resulting differential chemical profile.

### 2.5. Extracts of C. spicatum Are Efficient Antifungal Agents

All the tested spiked centaurium extracts showed significant antifungal potential (Table 5). Among them, Cs1, Cs5, Cs8, and Cs9 were in general highly effective antifungal agents against all the tested strains (MIC = 0.25 mg/mL). Cs1 extract, characterized with a high content of iridoid glucosides, was effective against *A. versicolor* and *P. funiculosum*, whereas Cs2, Cs4, Cs5, and Cs9 showed the best antifungal effect toward *P. funiculosum*. Extract Cs7 showed the same uniform activity against all the tested fungi with MICs of 0.5 mg/mL, whereas extracts Cs5, Cs6, and Cs9 were most effective against *Trichoderma viride*. Data obtained in this study regarding antifungal properties of *C. spicatum* extracts are in accordance with previously published data [19]. The antifungal activity of extracts of various *Centaurium* species pointed to this genus as a promising source of herbal antifungals [18,33]. Prominent antifungal activity of swertiamarin and gentiopicrin toward *Aspergillus* strains has been determined by other authors as well [18,33], which indicates that *C. spicatum* extracts may be used to inhibit the growth of these food-contaminating and mycotoxin-producing fungi.

### 2.6. Anticandidal Activity

The increasing resistance of *Candida* species to numerous antifungal agents may be attributed to the increasing use of these drugs in the rising number of people suffering from candidemia [34]. Medicinal plants, which contain numerous compounds with antifungal activity, may be used also for the development of new, efficient antifungal agents [35]. The results of the Cs1-Cs9 anticandidal activity are presented in Table 6. All the tested Cs extracts are able to inhibit growth of the tested yeasts, rather uniformly. The most sensitive strain was *C. parapsilosis* ATCC 22019 (MIC 0.25 mg/mL), followed by *C. albicans* 475/15 (MIC 0.5 mg/mL). Contrary to this, the most resistant strain was *C. krusei* H1/16 (MIC 4 mg/mL). Previous studies [19,36] have shown that methanol extracts of *C. spicatum* actively inhibit growth of *C. albicans*, which is consistent with our study. However, even though previous reports [37,38] demonstrated that different solvents affect the activity of *C. erythraea* toward *C. albicans*, this is the first comprehensive study comparatively analyzing the potential of nine different *C. spicatum* extracts to inhibit the growth of non-albicans species of *Candida*, i.e.,—*C. parapsilosis*, *C. krusei*, and *C. tropicalis*.

### 2.7. Antibiofilm Activity

Biofilm formation complicates the antimicrobial treatment and contributes to high morbidity and mortality rates, which makes it one of the most important virulence factors contributing to *Candida* pathogenesis [39]. Among yeast-like fungi of the *Candida* genus, almost all clinically relevant species form a biofilm [40]. As a physical barrier, biofilm provides structural integrity and protection to pathogens against environmental factors, which, as a consequence, results in the decreased success of antimicrobial treatment against *C. albicans* infections. Therefore, the identification of plant extracts that could prevent and disrupt biofilm formation is an inspiring research task.

Results regarding antibiofilm activity of Cs extracts are presented in Table 7. Among the nine extracts, two have been distinguished as the most efficient anticandidal agents and were chosen for the evaluation of antibiofilm activity—i.e., Cs2 and Cs6. Selected extracts have been tested for their biofilm–disruptive properties toward two *Candida* strains: *C. albicans* 475/15 and *C. parapsilosis* ATCC 22019 (Table 7). The MIC value of the extract Cs2 reduced the ability of both tested *Candida* species in a similar manner—36.29% for *C. albicans* 475/15 and 39.79% for *C. parapsilosis* ATCC 22019. Lower concentrations of extract Cs2 (0.5 MIC and 0.25 MIC) resulted in lower inhibitory activity, with the most promising results observed at 0.5 MIC against *C. albicans* 475/15 (34.75% inhibition).

As for the Cs6 extract, it reduced the biofilm formation ability of *C. albicans*. 475/15 and *C. parapsilosis* ATCC 22019 by 26.33% and 41.30%, respectively, using MIC values. Contrary to Cs2, lower concentrations of Cs6 had no inhibitory effect on *C. parapsilosis* ATCC 22019. Nevertheless, since there are no previous reports indicating the anti-biofilm potential of *C. spicatum* extracts, not even for other species belonging to this genus, we may highlight the importance and pioneering aspect of results obtained in our study.

### 2.8. Anti-Hyphal Activity

Unlike highly specialized pathogens that express a single major virulence factor, the opportunistic fungal pathogen *Candida albicans* has a repertoire of activities that contribute to its virulence [41]. Virulence factors of *C. albicans* generally include dimorphism, i.e., the ability of the fungus to grow in different morphological forms. As recently described [42], the formation of hyphae is a component of the overall virulence strategy of *C. albicans*. Therefore, the study of natural products with anti-hyphae activity is a relatively new strategy to combat the pathogenicity of *C. albicans*.

In the present study, both tested extracts, Cs2 and Cs6, were able to reduce the ability of yeast cells to form hyphae (Table 8); thus, we may say that the tested extracts inhibited the onset of virulence factors. In more detail, the treatment of *Candida* cells with Cs6 extract reduced the number of hyphae from 31.09% (untreated sample) to 9.09% (sample treated with Cs6).

### 2.9. Cytotoxicity and Effect of Extracts on the Migration Potential of HaCaT Cells

The wound scratch healing assay was used to test the migration potential of the HaCaT cell line (human keratinocyte cell line) after treatment with the studied extracts. Although all tested samples showed no toxicity (IC50 > 400 µg/mL) (Table 9), their wound healing potential differed significantly, both in terms of the control and tested sample.

Thus, the extracts showed the ability to heal wounds with a significantly higher percentage than the control (Table 10).

**Figure 3:**
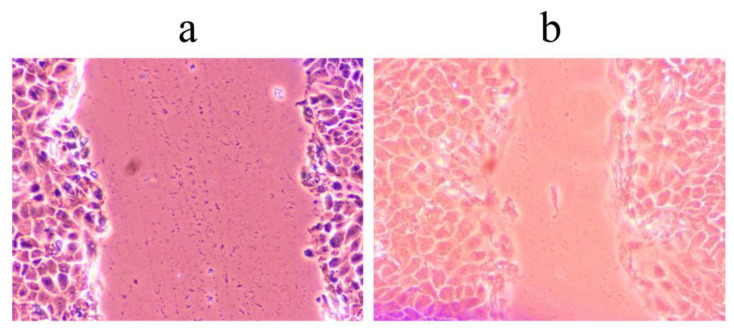
Representative images of wound closure assay in HaCaT cells treated with Cs3 extract at (**a**) the beginning of measurement (0 h) and (**b**) the final measurement (after 48 h) taken under magnification 10× and optical zoom 33%.

The most striking result of this study was the wound healing potential of Cs3 and Cs4 extracts with an efficiency of as much as 41.98% and 27.66%, respectively (Figure 3). Interestingly, the extracts Cs3 and Cs4 are among the extracts with high rutin content, which may indicate that the content of this compound in extracts could be associated with the migratory ability of HaCaT cells. Another species in the genus *Centaurium*, *C. erythraea*, has been recommended for external use in the treatment of wounds [43]. According to these findings and the wound closure induced by the mentioned extracts, this in vitro study could be a springboard for further investigations.

## 3. Materials and Methods

### 3.1. Plant Collection and Extractions

The aerial parts of *Centaurium spicatum* (30–60 cm) were collected in July 2021 near the beach of Buljarice (geographic coordinates: 42.19324, 18.96843) in Montenegro. Plant materials were collected and identified by the botanist authors (J.B., D.S., and D.M.) by using the scientific botanical literature and plant morphological features. The plant species was deposited in our local institutional herbarium under voucher number CSBCG2021. Plants were air-dried and cut into small pieces. The samples (2 g) were crushed with liquid nitrogen and then extracted with 20 mL of the solvents listed in Table 1. After 48 h at 4 °C, the samples were filtered through Whatman paper No. 4. The residue was re-extracted with an additional portion of solvent (20 mL) at 4 °C two more times for 24 h and 48 h, respectively. Totally, nine different extracts (Cs1-9) were prepared. Extracts Cs1, Cs6, Cs7, Cs8, and Cs9 were evaporated to dryness at 40 °C using a rotary vacuum evaporator (Büchi R-210), while the water-containing extracts (Cs2, Cs3, Cs4, and Cs5) were frozen at −20 °C and subsequently lyophilized (LH Leybold, Lyovac GT2, Frenkendorf). The extract yield was calculated using the following equation:

Yield (%) = 100 × m(ext)/m(pl)(1)

where m(ext) is the mass of the extract and m(pl) is the mass of the plant sample.

### 3.2. UHPLC/(±)HESI-QqQ-MS/MS Targeted Metabolomics Analysis

Separation and quantification of compounds in tested samples were performed using a Dionex Ultimate 3000 UHPLC system equipped with a TSQ Quantum Access Max triple-quadrupole (QqQ) mass spectrometer (ThermoFisher Scientific, Basel, Switzerland). Elution was performed at 30 °C on a Syncronis C18 column (100 mm × 2.1 mm) with a 1.7 μm particle size (ThermoFisher Scientific, Basel, Switzerland). The mobile phase consisted of (A) water with 0.1% formic acid (MS grade) and (B) acetonitrile with 0.1% formic acid (both MS grade), which were applied in the previously described gradient elution program [44]: 5% B in the first 2.0 min, 2.0–14.0 min 5–95% B, 14.0–14.2 min from 95% to 5% B, and 5% B until the 20th min. The flow rate was set to 0.3 mL/min, and the injection volume was 5 μL. All analyses were performed in triplicate.

The parameters of the TSQ Quantum Access Max QQQ mass spectrometer equipped with heated electrospray ionization (HESI) source were the same as previously described in Božunović et al. [18]: vaporizer temperature of 450 °C, spray voltage 4000 V, sheet gas (N2) pressure 50 AU, ion sweep gas pressure 0 AU and auxiliary gas pressure of 20 AU, capillary temperature at 320 °C, and skimmer offset 0 V. The MS data were acquired in negative ionization mode, in the m/z range from 100 to 1000. Multiple mass spectrometric scanning modes, including full scanning (FS), product ion scanning (PIS), and neutral loss scanning (NLS), were conducted for the qualitative analysis. Collision-induced fragmentation experiments were performed using argon as the collision gas, with collision energy set to 30 eV. A selected reaction monitoring (SRM) experiment for quantitative analysis was performed using two characteristic MS^2^ fragments for each targeted compound (Table S1), which were previously defined as dominant in PIS experiments [45].

### 3.3. Antioxidant Activity

#### 3.3.1. FRAP and ABTS Assays

Ferric-reducing antioxidant power (FRAP) and ABTS radical cation scavenging activity assays were performed as previously described in Božunović et al. [18].

Briefly, the working FRAP reagent contained 300 mM Na-acetate buffer (pH = 3.6), 20 mM ferric chloride, and 10 mM ferric-2,4,6-tri(2-pyridil)-1,3,5-triazine (Fe^3+^-TPTZ) at 10:1:1 ratio. Fe^3+^-TPTZ was dissolved in 40 mM HCl solution. Each sample was tested in triplicate by combining 950 μL FRAP reagent and 50 μL of the sample, followed by 10 min incubation at room temperature. Absorbance at 593 nm was determined with Agilent 8453 spectrophotometer (Agilent Technologies, Waldbronn, Germany). Methanol solutions of gallic acid (GA) were used for the construction of the calibration curve, and the results are expressed as GA equivalents reducing activity (mmol GAE) per 100 mg^−1^ plant extract. All analyses are performed in triplicate.

ABTS radical cations were obtained by combining equal volumes of 7 mM ABTS (2,20-azinobis(3-ethylbenzothiazoline-6-sulphonate)) and 2.45 mM potassium persulfate, after which the reagent was incubated in the dark at room temperature for 12 h. The solution was diluted with 80% ethanol until an absorbance of 0.7 ± 0.02 at 734 nm was obtained and used as the ABTS test reagent. Reaction mixtures containing 30 μL of sample and 970 μL of ABTS test reagent were incubated for 10 min at room temperature, and absorbance at 734 nm was subsequently measured. ABTS radical scavenging activity (%) was calculated using the formula:

ABTS^+^ activity = [(Acontrol − Asample)/Acontrol] × 100 (2)

where Asample represents the absorbance of the solution when the sample/reference compound has been added; Acontrol represents absorbance of the ABTS^+^ solution without extract added. Methanol solutions of gallic acid (GA) were used for the calibration curve construction and the results are presented as mmol GAE per 100 mg^−1^ plant extract. All analyses were performed in triplicate.

#### 3.3.2. EPR Determination of Scavenging Activity toward DPPH Radicals

The interaction of DPPH free radicals with antioxidant compounds in the extracts was evaluated by measuring the intensity of the DPPH EPR signal. The DPPH radical is EPR active, which makes this compound suitable for EPR assays. The distinctive shape of the DPPH EPR spectra enables the determination of the initial radical concentration and the ability of spiked centaury extracts to reduce the amount of DPPH in the system. Each extract dissolved in water (29 µL) was mixed with 3.2 mM DPPH solution (1 µL) and transferred to a 1 mm diameter Teflon tube. After 2 min, EPR spectra were recorded with the following parameters: Center field 3510 G, microwave power 10 mW, microwave frequency 9.85 GHz, modulation frequency 100 kHz, and modulation amplitude 2 G.

The anti-DPPH activity (AA) of the tested extracts was calculated using the formula [29]:

AA = ((Ic − Ia)/Ic) × 100 (%)(3)

where Ic and Ia refer to the double integral values of the control and samples, respectively, determined from the EPR spectra (using Xepr software). The calculated an-ti-DPPH activities of the extracts are shown in Figure 2.

#### 3.3.3. EPR Determination of the Scavenging Activity toward the Hydroxyl Radicals

To determine the capacity of *C. spicatum* extracts to remove hydroxyl radicals in the system, the solution consisting of the extract in the presence of a Fenton reaction with spin trap DEPMPO was used [46]. This spin trap was selected for its well-known suitable selectivity and long DEPMPO/OH spin-adduct half-life. Briefly, the reaction mixture (29 µL in total) consisting of sample the extract (26 µL), H_2_O_2_ (2 µL, final concentration 0.35 mM), and DEPMPO (1 µL; final concentration 3.5 mM) was transferred to the gas-permeable Teflon tube, after which FeSO_4_ (1 µL; final concentration 0.15 mM) was added immediately. Thereafter, EPR spectra were recorded 2 min later with the following experimental settings: center field 3500 G, microwave power 10 mW, microwave frequency 9.85 GHz, modulation frequency 100 kHz, modulation amplitude 1 G. The control experiments in both DPPH and ^•^OH radical scavenging activity tests have been made by replacing the sample with the same amount of solvent.

The anti-OH activity (AB) of the tested extracts was calculated using the formula [29]:

AB = ((Ic − Ia)/Ic) × 100 (%)(4)

where Ic and Ia refer to the double integral values of the control and samples, respectively, determined from the EPR spectra (using Xepr software). The calculated anti-OH activities of the extracts are shown in Figure 2.

### 3.4. Antibacterial and Antifungal Activities

The following Gram (+) bacteria: *Staphylococcus aureus* (ATCC 11632), *Bacillus cereus* (food isolate), and *Listeria monocytogenes* (NCTC 7973), and the Gram (−) bacteria *Escherichia coli* (ATCC 25922), *Enterobacter cloacae* (ATCC 35030), and *Salmonella* Typhimurium (ATCC 13311) were tested using microdilution method in a 96-well microtiter plate. Antifungal activity of extracts was evaluated against the following micromycetes: *Aspergillus fumigatus* (ATCC 9197), Aspergillus versicolor (ATCC 11730), *Penicillium funiculosum* (ATCC 36839), *Penicillium verrucosum var. cyclopium* (food isolate), *Trichoderma viride* (IAM 5061). The tested microorganisms are deposited in the Mycological Laboratory, Department of Plant Physiology, Institute for Biological Research “Sinisa Stanković”- National Institute of the Republic of Serbia, University of Belgrade, Serbia. Minimal inhibitory concentrations (MIC) and minimal bactericidal concentrations (MBC) of the tested extracts were determined using the modified CLSI 2009 protocol [47].

A modified microdilution technique was employed to examine the extracts’ antibacterial activity [47]. In the Luria broth medium, bacterial species were grown for 24 h at 37 °C. With sterile 0.85% saline that contained 0.1% Tween 80 (*v*/*v*), the fungal spores were collected from the surface of the agar plates that were previously inoculated with microfungi and grown for 21 days at 28 °C. Sterile saline was used to adjust the bacterial cells and fungal spore suspension to a concentration of roughly 1.0 × 10^5^ CFU in a final volume of 100 μL per well. For later use, the inocula were kept in storage at 4 °C. To ensure there was no contamination and to confirm the validity of the inoculum, dilutions of the inocula were cultured on Mueller-Hinton agar for bacteria and solid malt agar for fungi.

A serial dilution procedure was used to determine the minimum inhibitory concentration (MIC) in 96-well microtiter plates. The studied extracts were dissolved in 30% EtOH (20 mg/mL) and added to broth malt medium (for fungi) or Luria broth medium (for bacteria) with the inocula. The microplates were incubated for 24 h at 37 °C for bacteria and 72 h at 28 °C for fungi. The following day, 30 μL of INT (*p*-iodonitrotetrazolium violet) solution 0.2 mg/mL was added to the tested bacteria-containing medium, and the plates were placed back in the incubator for at least 30 min to guarantee a sufficient color reaction (Tsukatani et al., 2012). A clear solution or a distinct decrease in color response were indicators of growth inhibition. MICs were established as the lowest concentrations at which there was no discernible growth (under the binocular microscope). By serially subcultivating a 2 μL sample onto microtiter plates with 100 μL of broth in each well and then incubating the plates for 24 h or 72 h at 28 °C or 37 °C, the minimum bactericidal concentrations (MBCs) and minimum fungicidal concentrations (MFCs) were found. MBC/MFC stood for the lowest concentration with no discernible growth and represented a 99.5% killing of the original inoculum. The experiments were repeated in triplicate for each concentration used. E211 (sodium benzoate) and E244 (potassium metabisulfite) were used as positive controls. A 30% EtOH-negative control was employed. MIC and MBC/MFC values were expressed in mg/mL.

### 3.5. Anticandidal Assay

For the determination of minimum inhibitory (MIC) and minimum fungicidal concentrations (MFC), the microdilution method with some modification was used (EUCAST 2002). The following strains were used: *Candida albicans* ATCC 10231, *C. albicans* 475/15, *C. albicans* 1/6/15, *C. parapsilosis* ATCC 22019, *C. krusei* H1/16, and *C. tropicalis* ATCC 750. Yeast cultures were adjusted to McFarland 0.5 using sterile PBS (Phosphate-buffered saline). The 96-well microtiter plates containing serially diluted tested extracts (0.02–8.00 mg/mL) in liquid broth were incubated at 37 °C for 24 h. After incubation, the MIC and MFC were determined. The lowest concentrations without microscopically observed growth were considered MIC. For microscopic determination of growth, a Nikon Eclipse TS2 inverted microscope (Amsterdam, The Netherlands) was used, and fungal growth in the wells of 96-well microtiter plates was examined in comparison with the control (untreated yeast cells). MFC values were determined as concentrations without visible growth after serial subcultivation of 10 µL of the samples at 37 °C for 24 h. Ketoconazole (SigmaAldrich, Steinheim, Germany) was used as a positive control.

### 3.6. Antibiofilm Activity

*C. albicans* 475/15 and *C. parapsilosis* ATCC 22019 were incubated with MIC and sub-MIC of the selected extracts, Cs2 and Cs6, respectively, in YPD (Yeast Extract–Peptone–Dextrose) medium at 37 °C for 24 h. After incubation, the plate was washed twice with sterile PBS and fixed with methanol for 10 min. The methanol was removed, and the plate was air-dried. The residual biofilm was stained with 0.1% crystal violet (Sigma Aldrich, Steinheim, Germany) for 30 min. Subsequently, the plate was washed with water and air-dried, and the residual stain was dissolved with 96% ethanol (Zorka, Sabac, Serbia). Absorbance was measured at 620 nm on a Multiskan™ FC microplate photometer (Thermo Scientific™, Waltham, MA, USA), and results are presented as inhibition of biofilm formation (%) according to the equation:

[(A620control − A620sample)/A620control] × 100(5)

where A620control refers to the absorbance of the solution of untreated candida biofilm and A620sample refers to the absorbance of the solution of treated candida biofilm.

### 3.7. Anti-Hyphal Activity

*C. albicans* 475/15 was incubated with tested extracts for 4 h at 37 °C using YPD supplemented with 10% FBS (FBS, Gibco, Life Technologies Ltd., U.K.). After incubation, fungal cells were examined with a microscope (Nikon Eclipse TS2, Amsterdam, Netherlands). The number of cells that grew in the yeast or hyphal form was determined, and the percentage of hyphae was calculated according to Ivanov et al. [48] by the following equation:

Hyphae (%) = n(hyphae)/n(total) × 100

With n(hyphae) representing the number of cells growing in the hyphal morphology and n(total) representing the total number of *Candida* cells observed under a microscope.

### 3.8. Cytotoxicity toward HaCaT Cells

The cytotoxic effect of the extracts was determined using a spontaneously immortalized keratinocyte cell line (HaCaT, AddexBio T0020001) in the crystal violet assay, as previously described by Stojković et al. [49] with some modifications. HaCaT cells were grown in high-glucose Dulbecco’s Modified Eagle Medium (DMEM) supplemented with 10% fetal bovine serum (FBS, Gibco, Life Technologies Ltd., U.K.), 2 mM L-glutamine, and 1% antibiotic-antimycotic (Gibco, Life Technologies Corporation, Carlsbad, CA, USA) at 37 °C in a 5% CO_2_ incubator. Cells (1.7 × 104 cells/well) were seeded into a 96-well microtiter plate with an adhesive bottom. After 24 h, the medium was removed, and the cells were treated with different concentrations of the extracts for the next 24 h. Subsequently, the medium was removed, and cells were washed twice with PBS pH 7.4 (Sigma-Aldrich, St. Louis, MI, USA), after which they were stained with 0.4% crystal violet (CV, Sigma-Aldrich, St. Louis, MI, USA) staining solution for 20 min. The CV staining solution was removed, and the cells were washed in a stream of tap water and air-dried at room temperature. The absorbance of the dye dissolved in methanol was measured at 570 nm (OD570) in a microplate reader. The results were expressed as IC50 value indicating 50% cell viability compared to the untreated control. The criterion for grading the cytotoxicity of the extracts in the HaCaT cell line was as follows: IC50 ≤ 20 µg/mL = strongly cytotoxic, IC50 between 21 and 200 µg/mL = moderately cytotoxic, IC50 between 201 and 400 µg/mL = weakly cytotoxic, and IC50 > 401 µg/mL = no cytotoxicity. The solvent was used as a negative control.

IC = [(A570control − A570sample)/A570control] × 100(6)

### 3.9. Wound Scratch Healing Assay

The assay was performed as described by Đorđevski et al. [49] with some modifications. HaCaT cells were grown until reaching 85% confluence. The cell monolayer was scratched with a 200 μL sterile tip. Floating cells were washed, and cells were incubated in reduced DMEM supplemented with 1% FBS, 2 mM L-glutamine, and 1% antibiotic-antimycotic, containing 400 µg/mL of preparations. Cell migration was monitored using Nikon Eclipse TS2 (Amsterdam, the Netherlands) 48 h after wound preparation and treatment. The untreated control was used to measure wound closure under these conditions and without the addition of preparations. Results were presented as the percentage of wound closure during exposure to the tested extracts.

## 4. Conclusions

This study was the first to investigate the biological potential and chemical composition of *C. spicatum* as influenced by different extraction solvent systems. All nine *C. spicatum* extracts obtained within this study exerted various biological activities, including antimicrobial, antioxidant, anticandidal, antibiofilm, and wound healing. These biological activities can be altered and even enhanced by appropriate extraction procedures. In this study, we showed that among flavonoids, rutin was the most abundant compound in all analyzed extracts, and among iridoid compounds, sweroside predominated. The results indicate that ethanol-water-based extraction procedures (Cs2, Cs3, and Cs4) were the most efficient in isolating polyphenols. Consequently, the mentioned extracts proved to be the most suitable antioxidants. Moreover, the ethanol:water extracts Cs3 and Cs4 showed the best wound healing potential. Furthermore, Cs2 extract inhibited almost 40% of the preformed biofilm of *C. parapsilossis*, while butanol extract Cs6 was more efficient in reducing the ability of yeast cells to form hyphae. Considering the obtained results, we can conclude that solvent composition may be altered in a way to favor the extraction of desired compounds, or groups of compounds, from polyphenol- and iridoid-rich *C. spicatum*, depending on the biological activity needed to be exerted. Further in vivo studies are needed to confirm these results in order to make this plant commercially exploitable at higher levels.

## Figures and Tables

**Table 1:** *C. spicatum* extract used in this study.

Sample	Extraction Solvent	Yield of Extract (%)
**Cs1**	Ethanol 100%	24.0
**Cs2**	Ethanol:water 70:30	24.2
**Cs3**	Ethanol:water 50:50	25.0
**Cs4**	Ethanol:water 30:70	23.9
**Cs5**	Water 100%	23.2
**Cs6**	Butanol 100%	10.0
**Cs7**	Butanol:ethanol 70:30	17.8
**Cs8**	Butanol:ethanol 50:50	22.8
**Cs9**	Butanol:ethanol 30:70	29.5

**Table 2:** Polyphenolic and iridoid constituents detected in *C. spicatum* extracts.

mg/kg	Cs1	Cs2	Cs3	Cs4	Cs5	Cs6	Cs7	Cs8	Cs9
**Polyphenolics**									
**Aesculin**	7.33 ± 0.26	6.03 ± 0.1	5.53 ± 0.37	5.37 ± 0.05	5.21 ± 0.31	5.72 ± 0.08	6.57 ± 0.24	6.41 ± 0.18	4.85 ± 0.20
**Chlorogenic acid**	5.16 ± 0.15	5.04 ± 0.11	4.64 ± 0.25	5.39 ± 0.10	4.57 ± 0.33	5.18 ± 0.25	5.12 ± 0.16	5.64 ± 0.17	4.83 ± 0.23
**Aesculetin**	2.77 ± 0.05	2.36 ± 0.19	3.50 ± 0.14	2.07 ± 0.03	1.79 ± 0.29	3.74 ± 0.17	3.52 ± 0.25	3.34 ± 0.13	2.13 ± 0.07
**Caffeic acid**	7.94 ± 0.32	20.68 ± 1.17	18.74 ± 0.58	17.53 ± 0.44	**17.51 ± 0.59**	7.22 ± 0.16	6.55 ± 0.46	6.97 ± 0.33	5.95 ± 0.26
**Rutin**	**76.45 ± 0.44**	**125.25 ± 3.35**	**109.48 ± 2.29**	**96.38 ± 3.81**	3.32 ± 0.12	**39.49 ± 1.25**	**48.94 ± 0.53**	**56.10 ± 2.02**	**59.03 ± 1.54**
***p*-Coumaric acid**	NF	NF	NF	NF	7.33 ± 0.21	3.07 ± 0.32	NF	NF	NF
**Naringin**	6.46 ± 0.20	4.39 ± 0.27	4.83 ± 0.43	2.20 ± 0.27	1.25 ± 0.19	13.35 ± 0.32	12.33 ± 0.60	8.37 ± 0.19	10.83 ± 0.20
**Astragalin**	1.16 ± 0.04	0.79 ± 0.04	0.84 ± 0.04	0.64 ± 0.04	1.00 ± 0.04	1.44 ± 0.06	1.37 ± 0.07	1.39 ± 0.09	0.99 ± 0.08
**Rosmarinic acid**	0.91 ± 0.06	NF	0.78 ± 0.07	2.56 ± 0.04	NF	NF	1.05 ± 0.03	NF	0.92 ± 0.04
**Aromadedrin**	3.76 ± 0.22	1.04 ± 0.02	2.39 ± 0.06	NF	NF	4.64 ± 0.48	3.99 ± 0.29	3.32 ± 0.28	3.71 ± 0.20
**Eriodictyol**	8.12 ± 0.06	8.36 ± 0.12	9.51 ± 0.30	9.69 ± 0.36	7.52 ± 0.37	6.98 ± 0.44	9.43 ± 0.33	7.03 ± 0.32	7.76 ± 0.18
**Luteolin**	0.94 ± 0.05	0.40 ± 0.06	0.69 ± 0.08	NF	NF	1.20 ± 0.10	0.94 ± 0.04	0.91 ± 0.07	0.57 ± 0.10
**Quercetin**	14.32 ± 0.23	11.58 ± 0.1	10.89 ± 0.37	NF	NF	20.00 ± 0.50	19.58 ± 0.49	17.70 ± 0.52	16.64 ± 0.06
**Naringenin**	NF	NF	NF	NF	NF	2.08 ± 0.12	1.43 ± 0.10	1.47 ± 0.11	0.77 ± 0.05
**Isorhamnetin**	1.77 ± 0.03	1.18 ± 0.03	1.62 ± 0.06	0.94 ± 0.08	0.56 ± 0.06	3.01 ± 0.01	2.82 ± 0.29	2.68 ± 0.17	2.43 ± 0.18
**SUM**	137.08	**187.10**	173.45	142.78	50.05	117.13	123.64	121.33	121.40
**Iridoids**									
**Loganic acid**	0.32 ± 0.05	0.37 ± 0.05	0.31 ± 0.04	0.40 ± 0.06	0.55 ± 0.08	0.18 ± 0.03	0.28 ± 0.04	0.35 ± 0.05	0.27 ± 0.04
**Swertiamarin**	18.55 ± 2.35	15.62 ± 1.98	10.64 ± 1.35	11.90 ± 1.51	10.43 ± 1.32	10.53 ± 1.34	9.30 ± 1.18	9.06 ± 1.15	9.22 ± 1.17
**Gentiopicrin**	15.18 ± 0.74	10.37 ± 0.50	13.14 ± 0.64	7.13 ± 0.35	7.17 ± 0.35	11.37 ± 0.55	17.43 ± 0.85	17.23 ± 0.84	12.31 ± 0.60
**Sweroside**	**50.41 ± 2.39**	**48.08 ± 2.28**	**36.21 ± 1.72**	**55.33 ± 2.62**	**45.53 ± 2.16**	**62.89 ± 2.98**	**62.28 ± 2.95**	**57.34 ± 2.72**	**51.17 ± 2.43**
**SUM**	84.46	74.44	60.30	74.76	63.68	**84.97**	89.29	83.98	72.97

The values are expressed as mean ± SD in mg/kg of plant dry weight. NF-not found.

**Table 3:** Polyphenolic and iridoid constituents detected in *C. spicatum* extracts. Letters above the bars denote significant differences according to Fisher’s LSD test at *p* ≤ 0.05. Samples denoted by the same letter are not significantly different.

Extract	ABTS mmol GAE per 100 mg Extract	FRAP mmol GAE per 100 mg Extract
**Cs1**	23.46 ± 0.45 ^a^	45.40 ± 1.07 ^a^
**Cs2**	37.74 ± 0.74 ^b^	55.13 ± 0.62 ^b^
**Cs3**	36.76 ± 0.55 ^b^	53.84 ± 1.29 ^cd^
**Cs4**	34.47 ± 0.63 ^c^	50.99 ± 0.38 ^d^
**Cs5**	21.39 ± 0.23 ^d^	2.70 ± 0.85 ^e^
**Cs6**	29.75 ± 0.55 ^e^	62.41 ± 1.30 ^bc^
**Cs7**	27.42 ± 0.77 ^f^	54.20 ± 1.34 ^d^
**Cs8**	27.18 ± 0.74 ^f^	47.90 ± 1.43 ^a^
**Cs9**	24.62 ± 0.69 ^a^	48.51 ± 1.11 ^a^

**Table 4:** Antibacterial activity of *C. spicatum* extracts mg/mL. For positive controls, E211—sodium benzoate and E244—potassium metabisulfite were used.

		*Staphylococcus aureus*	*Bacillus cereus*	*Listeria monocytogenes*	*Salmonella Typhimurium*	*Escherichia coli*	*Enterobacter cloacae*
		(ATCC 11632)	(Clinical Isolate)	(NCTC 7973)	(ATCC 13311)	(ATCC 25922)	(ATCC 35030)
**Cs1**	MIC	1.0	1.0	0.5	2.0	2.0	2.0
	MBC	2.0	2.0	1.0	4.0	4.0	4.0
**Cs2**	MIC	1.0	1.0	1.0	2.0	2.0	2.0
	MBC	2.0	2.0	2.0	4.0	4.0	4.0
**Cs3**	MIC	1.0	1.0	2.0	2.0	2.0	2.0
	MBC	2.0	2.0	4.0	4.0	4.0	4.0
**Cs4**	MIC	0.5	0.5	2.0	2.0	2.0	2.0
	MBC	1.0	1.0	4.0	4.0	4.0	4.0
**Cs5**	MIC	1.0	0.5	1.0	2.0	1.0	2.0
	MBC	2.0	1.0	2.0	4.0	2.0	4.0
**Cs6**	MIC	1.0	1.0	2.0	2.0	2.0	2.0
	MBC	2.0	2.0	4.0	4.0	4.0	4.0
**Cs7**	MIC	1.0	1.0	1.0	2.0	1.0	2.0
	MBC	2.0	2.0	2.0	4.0	2.0	4.0
**Cs8**	MIC	0.5	1.0	2.0	1.0	1.0	1.0
	MBC	1.0	2.0	4.0	2.0	2.0	2.0
**Cs9**	MIC	2.0	1.0	2.0	1.0	2.0	1.0
	MBC	4.0	2.0	4.0	2.0	4.0.	2.0
**E211**	MIC	4.0	0.5	1.0	1.0	1.0	2.0
	MBC	4.0	0.5	2.0	2.0	2.0	4.0
**E224**	MIC	1.0	2.0	0.5	1.0	0.5	0.5
	MBC	1.0	4.0	1.0	1.0	1.0	0.5

**Table 5:** Antifungal activity of *C. spicatum* extracts mg/mL. For positive controls, E211—sodium benzoate and E244—potassium metabisulfite were used.

		*Aspergillus fumigatus*	*Aspergillus versicolor*	*Penicillium funiculosum*	*Penicillium verrucosum* var. *cyclopium*	*Trichoderma viride*
		(ATCC 9197)	(ATCC 11730)	(ATCC 36839)	(Food Isolate)	(IAM 5061)
**Cs1**	MIC	0.50	0.25	0.25	0.50	0.50
	MFC	1.00	0.50	0.50	1.00	1.00
**Cs2**	MIC	0.50	0.50	0.25	1.00	0.50
	MFC	1.00	1.00	0.50	2.00	1.00
**Cs3**	MIC	1.00	0.50	0.50	0.50	0.50
	MFC	2.00	1.00	1.00	1.00	1.00
**Cs4**	MIC	0.50	0.50	0.25	1.00	0.50
	MFC	1.00	1.00	0.50	2.00	1.00
**Cs5**	MIC	0.50	0.50	0.25	0.50	0.25
	MFC	1.00	1.00	0.50	1.00	0.50
**Cs6**	MIC	0.50	0.50	0.50	0.50	0.25
	MFC	1.00	1.00	1.00	1.00	0.50
**Cs7**	MIC	0.50	0.50	0.50	0.50	0.50
	MFC	1.00	1.00	1.00	1.00	1.00
**Cs8**	MIC	0.25	0.25	0.50	0.50	0.50
	MFC	0.50	0.50	1.00	1.00	1.00
**Cs9**	MIC	0.50	0.50	0.25	0.50	0.25
	MFC	1.00	1.00	0.50	1.00	0.50
**E211**	MIC	1.00	2.00	1.00	2.00	1.00
	MFC	2.00	4.00	2.00	4.00	2.00
**E224**	MIC	1.00	1.00	0.50	1.00	0.50
	MFC	1.00	1.00	0.50	1.00	0.50

**Table 6:** Anticandidal activity of examined extracts, results are in mg/mL.

		Cs1	Cs2	Cs3	Cs4	Cs5	Cs6	Cs7	Cs8	Cs9	Ketoconazole
***C.albicans* ATCC 10231**	MIC	1.000	1.000	1.000	1.000	1.000	1.000	1.000	1.000	1.000	0.002
	MFC	2.000	2.000	2.000	2.000	2.000	2.000	2.000	2.000	2.000	0.006
***C.albicans* 475/15**	MIC	0.500	0.500	0.500	0.500	0.500	0.500	0.500	0.500	0.500	0.003
	MFC	1.000	1.000	1.000	1.000	1.000	1.000	1.000	1.000	1.000	0.006
***C.albicans* 1/6/15**	MIC	2.000	2.000	2.000	2.000	2.000	2.000	2.000	2.000	2.000	0.003
	MFC	4.000	4.000	4.000	4.000	4.000	4.000	4.000	4.000	4.00	0.006
***C.parapsilosis* ATCC 22019**	MIC	0.250	0.250	0.250	0.250	0.250	0.250	0.250	0.250	0.250	0.003
	MFC	0.500	0.500	0.500	0.500	0.500	0.500	0.500	0.500	0.500	0.006
***C. krusei* H1/16**	MIC	4.000	4.000	4.000	4.000	4.000	4.000	4.000	4.000	4.000	0.002
	MFC	8.000	8.000	8.000	8.000	8.000	8.000	8.000	8.000	8.000	0.003
***C.tropicalis* ATCC 750**	MIC	2.000	2.000	2.000	2.000	2.000	2.000	2.000	2.000	2.000	0.002
	MFC	4.000	4.000	4.000	4.000	4.000	4.000	4.000	4.000	4.000	0.006

**Table 7:** Antibiofilm activity of *C. spicatum* extracts, results are expressed as a percentage of inhibition of biofilm formation.

	Cs2			Cs6		
	MIC	0.5 MIC	0.25 MIC	MIC	0.5 MIC	0.25 MIC
***C.albicans* 475/15**	36.29 ± 6.58	34.75 ± 13.51	19.98 ± 10.32	26.33 ± 3.83	16.85 ± 12.78	1.47 ± 0.99
***C.parapsilosis* ATCC 22019**	39.79 ± 7.33	29.75 ± 6.15	1.59 ± 0.25	41.30 ± 1.94	* NE	NE

* NE—no effect.

**Table 8:** Percentage of hyphal cells calculated by comparison with the total number of *C. albicans* cells. Values are means ± SD of three replicates.

Extract	Percentage of Hyphae
**Cs2**	16.49 ± 1.89
**Cs6**	9.09 ± 2.01
**Control**	31.09 ± 2.22

**Table 9:** Cytotoxic activity of preparation toward the HaCaT cell line.

Samples	IC_50_ (µg/mL)
**Cs1**	>400
**Cs2**	>400
**Cs3**	>400
**Cs4**	>400
**Cs5**	>400
**Cs6**	>400
**Cs7**	>400
**Cs8**	>400
**Cs9**	>400

**Table 10:** Relative percentage of cells that migrated into the wound (%).

Samples	Wound Healing (%)
**Control**	0.083 ± 0.008
**Cs1**	10.210 ± 1.110
**Cs2**	14.230 ± 1.070
**Cs3**	41.980 ± 2.130
**Cs4**	27.660 ± 1.990
**Cs5**	15.130 ± 2.150
**Cs6**	8.510 ± 0.950
**Cs7**	9.930 ± 0.170
**Cs8**	6.210 ± 0.910
**Cs9**	7.580 ± 1.710

## Data Availability

All relevant data in the current study are available from the corresponding author on request.

## Supplementary Materials

The following supporting information can be downloaded at: https://www.mdpi.com/article/10.3390/ph16020245/s1, Table S1: List of polyphenolics in negative ion mode with the mass of parent ion (*m/z*) and masses of product ions (*m/z*) with specified collision energy (eV).
