# Supplementary material for: Solvent System-Guided Extraction of Centaurium spicatum (L.) Fritch Provides Optimized Conditions for the Biological and Chemical Characteristics of the Herbal Extracts
Source: Pharmaceuticals (Basel). 2023 Feb 6;16(2):245. doi: 10.3390/ph16020245 (PMC9967942; doi:10.3390/ph16020245)
Supplement: Supplementary file 1 [file pharmaceuticals-16-00245-s001.zip › pharmaceuticals-2160493-supplementary.pdf]

**Table S1**

| | Parent Ion, *m/z* | Product Ion, *m/z* (Collision Energy, *eV*) |
|---|---|---|
| | *Polyphenolics* | |
| **Aesculin** | 339.080 | 133.09(44); 177.06(25) |
| **Chlorogenic acid** | 353.103 | 191.28(25) |
| **Aesculetin** | 176.992 | 150.25(20); 133.28(19) |
| **Caffeic acid** | 179.004 | 134.00(13); 135.00(16) |
| **Rutin** | 609.197 | 299.98(42); 301.20(32) |
| ***p*-Coumaric acid** | 163.031 | 93.12(39); 119.09(16) |
| **Naringin** | 579.241 | 151.42(43); 217.26(33) |
| **Astragalin** | 447.008 | 284.03(29); 255.03(43) |
| **Rosmarinic acid** | 359.061 | 133.03(43); 161.00(21) |
| **Aromadedrin** | 287.009 | 269.00(15); 259.00(30) |
| **Eriodictyol** | 286.974 | 135.02(22); 150.93(19) |
| **Luteolin** | 285.035 | 133.06(36); 151.03(28) |
| **Quercetin** | 301.026 | 151.01(22); 179.00(20) |
| **Naringenin** | 271.036 | 151.01(20); 107.07(26) |
| **Isorhamnetin** | 315.040 | 300.03(30); 151.02(20) |
| | *Iridoids* | |
| **Loganic acid** | 374.620 | 212.85(20); 168.84(20) |
| **Swertiamarin + HCOOH** | 419.000 | 178.94(20); 118.79(20) |
| **Gentiopicrin + HCOOH** | 401.000 | 179.05(20); 148.77(20) |
| **Sweroside + HCOOH** | 403.000 | 194.70(20); 125.00(20) |
